# Malicious Tumor? Pathological Fracture of the Femur in Children Caused by Myelolipoma: A Case Report and Review of Literatures

**DOI:** 10.1155/2024/5838618

**Published:** 2024-05-15

**Authors:** Xiaoyu Shen, Qiang Yao, Xiangbei Qi, Lijie Ma

**Affiliations:** ^1^Department of Orthopaedic Surgery, Hebei Medical University Third Hospital, Shijiazhuang 050035, China; ^2^Internal Medicine, The First Hospital of Hebei Medical University, Shijiazhuang 050000, China

## Abstract

Myelolipoma is a kind of benign lipoma containing myeloid cells. It is a rare type of tumor that typically presents as an occasional adrenal tumor, generally manifesting as a nonfunctional adrenal mass. Although it can occur in extra-adrenal tissues, its occurrence in bone tissue is extremely rare. Most cases are discovered accidentally during physical examinations of adults, and there are currently no reports of cases with pathological fractures as the main symptoms. We present a case of a 15-year-old teenager who developed a pathological fracture caused by femoral myelolipoma. The diagnosis of the specific type of bone tumor of the patient was determined through pathology and imaging. To treat the condition, we utilized a technique known as the “soft drill” to fully access the tumor space, remove the bone septum, and scrape away the diseased tissue. The fracture was then stabilized using a hybrid external fixation. After a 2-year follow-up period, there was no recurrence of the bone tumor. This case is the first case of intraosseous myelolipoma that occurred in a minor with the initial symptom of pathological fracture, filling the gap in our existing body of knowledge and providing a reference for the treatment of this type of intraosseous myelolipoma.

## 1. Introduction

Myelolipoma is a rare benign tumor typically found in the adrenal gland [1]. In some cases, it can also occur in extra-adrenal tissues, including the head, thorax, abdomen, pelvis, spinal, paravertebral, and lymphonodal, such as the mandible, mediastinum, presacral, lung, liver, and spleen [2–6]. The youngest patient with extra-adrenal myelolipoma was reported by Adetiloye, Adejuyigbe, and Adelusola, which was a 1.5-year-old boy diagnosed with presacral myelolipomas with a history of urinary retention and constipation [7]. In most cases, it presents as an asymptomatic adrenal mass [8]. However, it can also cause symptoms by compressing surrounding organs and tissues [9]. It is extremely rare for myelolipoma to occur in bone tissue. In bones, it can also lead to fractures [10]. The previously reported cases were all adults with no fractures; therefore, the best treatment method is also unknown. Here, we have treated a teenager with a femoral shaft fracture caused by intraosseous myelolipoma. By sharing the process of diagnosis, surgery, and follow-up, we hope to enhance clinicians' understanding of the disease and provide a reference for the treatment of this disease.

## 2. Case Presentation

### 2.1. Chief Complaints and Physical Examinations

The patient was a 15-year-old teenager with a left thigh injury. He accidentally hurt his left thigh while descending the stairs at school. After examination, it was found that his left thigh was visibly swollen, with noticeable local tenderness and palpable bone rubbing. However, the skin was intact, and there were no signs of ulceration.

### 2.2. Laboratories and Examinations

**Figure 1:**
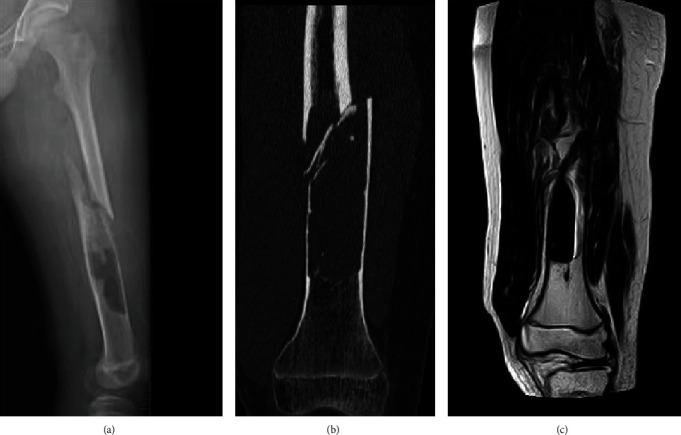
Imaging manifestations of myelolipoma. (a) X-ray showed a pathological fracture of the left femoral shaft. (b) CT showed the disappearance of bone trabeculae at the tumor and thin cortex. (c) MRI showed thinning of the bone cortex in the middle and lower segments of the left femur and patchy low signal shadow in the medullary cavity.

The laboratory examinations were essentially normal. The x-ray film showed a cystic space-occupying lesion in the middle and lower segments of the left femur, accompanied by a pathological fracture (Figure 1(a)). The CT scan showed that the boundary of the lesion was unclear. The bone cortex appeared thinner and more discontinuous, and a dense shadow of soft tissue was visible in the medullary cavity. No trabecular bone was found (Figure 1(b)). The signal in the medullary cavity was uneven on the MRI, and flake-like low signal shadows of various sequences were seen in the cavity (Figure 1(c)).

### 2.3. Final Diagnosis and Treatments

**Figure 2:**
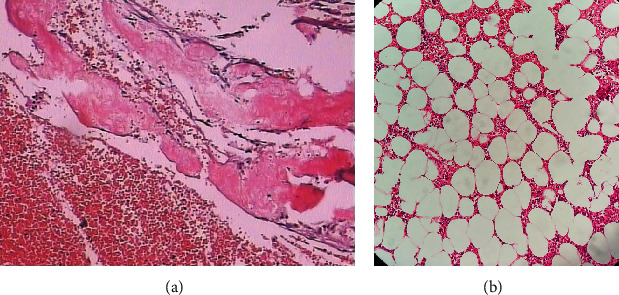
Pathology of myelolipoma. (a) Massive infiltration of acute and chronic inflammatory cells (40×). (b) Adipose tissue and bone marrow tissue can be seen in the tumor (100×).

The patient was diagnosed with a pathological fracture in the middle and lower segments of the left femur prior to the operation. The fracture was believed to be caused by either bone fibrous dysplasia or a bone cyst. We performed a biopsy on the site of the pathological fracture. Pathological sections showed that there was a massive infiltration of acute and chronic inflammatory cells in the tumor (Figure 2(a)). The tumor was composed of mature adipose tissue and hematopoietic cells. Hematopoietic cells were distributed among adipose tissue, including granulocytes, erythrocytes, and megakaryocytes, and the morphology and proportion of these cells were normal (Figure 2(b)). After consulting with the pathologists, it was diagnosed as femoral myelolipoma. The patient underwent a curettage procedure to remove the lesion. The fracture was fixed with a hybrid external fixation.

### 2.4. Surgery Processes

**Figure 3:**
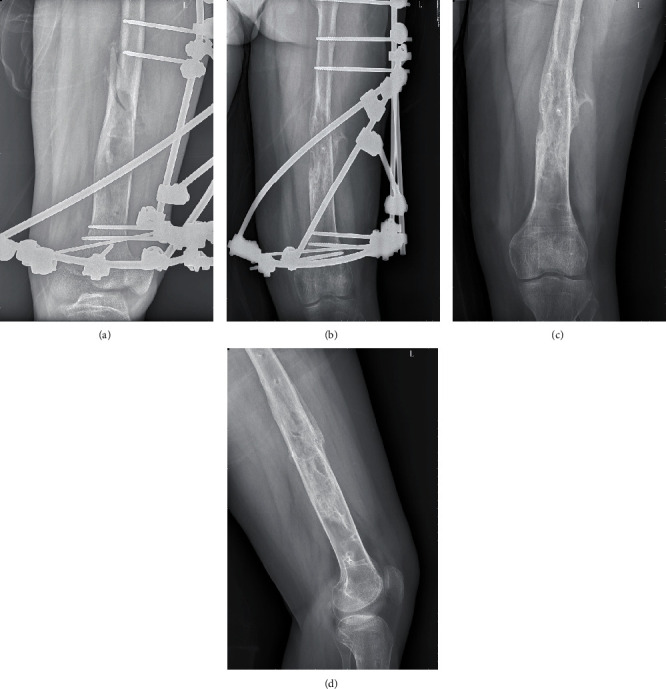
Postoperative x-ray and follow-up. (a) Initial postoperative day, featuring a multiplanar external frame for stabilization. (b) One-week postsurgery, where a secure external fixation is present without any displacement at the fracture site. (c, d) A 2-year follow-up period after surgery, indicating no recurrence.

During the operation, a lateral incision was made on the distal femur, with the fracture as the focal point. The skin, subcutaneous tissue, and iliotibial bundles were cut successively to gain access to the fracture end through the posterior space of the lateral femoral muscle. The blood clot and abnormal hyperplastic tissue at the fracture site were removed. The “soft drill” was adopted to fully open the tumor space and bone septum and scrape off the diseased tissue. Then, the medullary cavity was rinsed with a large amount of normal saline using a flushing gun. Considering the thin cortex of the fracture site in the distal femur, neither plate fixation nor elastic intramedullary nail fixation can provide sufficient stability. Additionally, there is no Ilizarov external fixation device available with a suitable diameter due to the patient's obesity. Therefore, the fracture was fixed with a hybrid external fixation (Figure 3(a)). In consideration of the rapid bone growth, robust fracture healing capacity, rejection reactions, and postoperative infections associated with bone grafting in adolescents and children, we made the decision not to utilize bone or other materials at the site of the bone defect.

### 2.5. Subsequent Treatments and Follow-Ups

After regular review, the fracture site was stable without obvious displacement (Figures 3(a) and 3(b)). After the fracture healing, the external fixation device for the pathological fracture of the left femoral shaft was removed under spinal anesthesia on November 11, 2019. The outpatient reexamination is conducted once every 6 months. As of June 2021, there was no recurrence (Figures 3(a) and 3(d)).

This case of pediatric intraosseous medullary lipoma was treated with comprehensive approaches, including reaming, opening the tumor space, flushing, and using hybrid external fixation. There was no recurrence after 2 years of follow-up.

## 3. Discussion

This is a case of a teenager diagnosed with femoral myelolipoma, with a pathological fracture as the initial symptom. Myelolipoma is a benign tumor composed of mature adipose tissue and hematopoietic cells, and the adrenal gland is the most common site of attack. In a few cases, it can also occur in extra-adrenal tissues, such as the presacral region, mediastinum, pelvic cavity, lung, liver, and spleen, of which the sacral anterior region is the most common [11, 12]. To our knowledge, there have been fewer than 60 reported cases of extra-adrenal myelolipoma, predominantly affecting females and typically discovered between the ages of 50 and 70 [13]. Primary myelolipoma of the bone is extremely rare, and only 8 cases have been reported when searching the Medline database [14]. The reported patients were all adults, and the affected areas included the femur, humerus, ilium, mandible, etc. The clinical manifestation of most patients is chronic pain in the affected area. Only one case report has described pathological fracture associated with myelolipoma, and the patient had systemic lupus erythematosus, antiphospholipid syndrome, and hyperparathyroidism [14], suggesting that it was not directly caused by the myelolipoma, while our case is a teenager with no clinical symptoms before the disease, with pathological fracture as the initial symptom, which is different from previously reported cases. Therefore, our case report may be the first to present an intraosseous myelolipoma with pathological fracture as the predominant symptom and can make a contribution to a better understanding of intraosseous myelolipoma.

Several etiologies have been proposed for myelolipomas, including the degeneration of hyperplastic nodules, adrenal adenomas, or myeloid mesenchymal cell hyperplasia. Overall, the most accepted hypothesis is that metaplasia occurs in the reticuloendothelial cells of the blood capillaries after a stressful stimulus [14, 15]. For intraosseous myelolipoma, abnormal differentiation of mesenchymal stem cells under pressure events may be one of the etiologies. It has been reported in the literature that bone lesions will eventually result in steatosis [16, 17]. There is a case report that suggested a long history of chronic autoimmune disease and that secondary hyperparathyroidism may have triggered the disease [14]. In our case, changes in the local internal environment of the bone tissue due to obesity and metabolic abnormalities may be the main cause.

Due to the rarity of myelolipoma and the majority of cases being asymptomatic, there is a scarcity of comprehensive studies on its detailed clinical features. Typically, physical examinations and routine blood tests do not yield any definitive diagnostic results. Depending on the size and location of the lesion, some patients may present with nonspecific lumbar or abdominal pain due to hemorrhage, mechanical compression, or tumor infarction [18]. The rarity of myelolipoma in bones is likely due to its lack of early manifestation as with other bone diseases. CT and MRI can assist in diagnosis; however, myelolipoma lacks characteristic imaging findings, making its differentiation solely based on imaging extremely challenging, which leads to a high rate of misdiagnosis. Its correct diagnosis relies on a combination of pathology, imaging, and clinical features and needs to be distinguished from several diseases [19]. Especially in cases of intraosseous myelolipoma accompanied by pathological fractures, the fracture often leads to hemorrhage and inflammatory reaction around the lesion, which destroys the structural organization of the diseased tissue and alters imaging signals. Consequently, a definitive diagnosis often relies on pathological examination. The histological characteristics of intraosseous myelolipoma are similar to those of adrenal myelolipoma and extra-adrenal myelolipoma, consisting of morphologically normal hematopoietic tissue and mature adipose tissue, with slight variations in the degree of hematopoietic cell proliferation while being slightly distinct from normal bone marrow tissue. Atrophy and the disappearance of bone trabeculae are characteristic morphological changes [20]. The imaging features of intraosseous myelolipomas are low-density osteolytic lesions with a clear border, which may be accompanied by irregular sclerosis in the surrounding area, but the cortical bone is not damaged or deformed, and usually with no periosteal reaction. However, Sakai et al. reported a rare case of myelolipoma in the distal femur metaphysis with massive extraskeletal lesions and periosteal reactions, suggesting that it should be distinguished from malignant bone tumors [21]. The biopsy findings of intraosseous myelolipoma can exhibit similarities to focal hematopoietic hyperplasia (FHH). However, in contrast to FHH, which typically manifests as an expansive osteolytic lesion with internal calcification, a classical myelolipoma radiologically appears as an osteolytic or osteosclerotic intramedullary lesion without cortical expansion, as reported in the available literature [22]. Intraosseous lipoma is also one of the rarest bone tumors, and it mostly occurs in the lower limbs. It manifests as regular or irregular morphology, single fat density, or mixed density areas dominated by fat on CT scan, characterized by calcification within the lesion and osteosclerosis at the edge of the lesion. Its histologic examination can show different stages of lipocyte involution but generally does not show any hematopoietic cells [23]. Fibrous dysplasia of the bone exhibits a spectrum of imaging manifestations, ranging from cystic to sclerotic and mixed patterns, typically displaying a “ground-glass” appearance. Diagnosing this condition solely based on a single imaging modality can be challenging due to its variable presentation. Fibrous dysplasia of the bone can be distinguished from intramedullary lipoma through clinical manifestations. Clinically, it is frequently associated with “pseudarthrosis” arising from pathological fractures and may also involve extraskeletal system components [24]. Other differential diagnoses to consider include melorheostosis. Melorheostosis is also a rare benign bone disease, characterized by bone hypertrophy, dysplasia, and sclerosis. The dripping candle wax sign of melorheostosis stems from the sclerosis of one side of the cortex and appears along the long axis of the bone. The most common position affected by melorheostosis is the long bones of the limbs and the auxiliary skeleton [25]. The differential diagnosis is shown in Table 1. The identification of these diseases mainly relies on imaging and pathological features, but in cases where the patient has a fracture, the final diagnosis ultimately depends on pathological results.

In this case, the patient was followed up for 2 years without recurrence, which is consistent with previous literature records. Myelolipoma is a benign lesion, and its onset is closely related to pressure events. Consequently, distinguishing between recurrence and new onset of the disease during long-term follow-up can be challenging. Simple x-rays can facilitate early detection. The treatment of this disease can only be achieved by destroying and cleaning up the original tissue structure. In addition to necessary surgery, we can also treat patients by addressing metabolic abnormalities, such as obesity, and correcting the local abnormal internal environment. Intraosseous myelolipoma is a kind of nonfunctional benign tumor and usually has a good prognosis. For the management of myelolipomas, the AACE/AAES guideline issued in 2009 recommended radiological reevaluation at 3 and 6 months and then annually for 1–2 years [26]. Although there has been no relevant literature reporting a case of malignant transformation of intraosseous myelolipoma, it can still lead to severe consequences such as fractures. We plan to continue follow-up with annual imaging exams to monitor for any recurrence of the patient.

## 4. Future Directions

Intraosseous myelolipoma has an extremely low incidence rate with an unknown etiology. Therefore, a greater number of case reports is imperative to elaborate on the specific characteristics of this lesion and establish more reliable and consistent diagnostic criteria alongside treatment protocols, which are based on the cooperation of multiple centers. Advanced gene sequencing technology can be used to explore the pathogenesis and detect key biomarkers to assist in diagnosis. Establishing animal models through techniques such as gene knockout or the induction of mutations may be beneficial for related research.

## 5. Conclusion

We presented an adolescent patient with intraosseous myelolipoma. We hope to enhance clinicians' understanding of the disease and provide a reference for the treatment of this disease through detailed case data, diagnosis, and treatment process.

## Figures and Tables

**Table 1:** Differential diagnosis of intraosseous lipoma.

**Disease**	**Symptoms or signs**	**Imaging features**	**Pathologic findings**
Adrenal myelolipoma and extra-adrenal myelolipoma	Asymptomatic, compression, and hemorrhage due to mass effect	Well-defined lesions with variable amounts of fat signals interspersed with high-density, intensifiable myeloid components	Normal adipose tissue and hematopoietic tissue
Intraosseous myelolipoma	Asymptomatic or associated with chronic pain, rarely pathological fractures	Intramedullary lytic lesions without cortical expansion, signal intensity similar to adrenal myelolipoma, and irregular sclerosis may be present	Normal adipose tissue and hematopoietic tissue
Localized osseous hematopoiesis	Asymptomatic or compression due to mass effect	Well-defined, expanded, lytic masses with linear high-density areas and calcifications	Normal bone marrow tissue
Intraosseous lipoma	Asymptomatic, chronic pain, and occasional pathological fractures	Local density similar to normal fat, well-defined borders, trabecular bone resorption, possible calcifications within the cavity, sparing the bone cortex, and no periosteal reaction	Normal adipose tissue and no hematopoietic tissue
Fibrous dysplasia of bone	Limb swelling, deformity, pathological fractures, and pain	Variable imaging features, mostly expansive lesions; bone enlargement or thickening; bending deformities of the affected long bones; thinned but intact cortex; and no periosteal reaction. Ground-glass or loofah-like changes	Abnormal proliferating fibrous tissue and immature trabecular bone
Waxy bone disease	Limb pain, bone and joint deformities, limited range of motion, and palpable irregularity of the bone surface	Imaging shows proliferative bone along the long axis of the bone, resembling a waxy appearance	Mixed composition of laminar and woven bone

## Data Availability

The patient imaging and pathological data used to support the findings of this study are included within the article. Other data can be achieved from the corresponding author upon request.
